# Feedback literacy for EFL speaking among first- and second-year Chinese English majors: structural associations and reported action strategies

**DOI:** 10.3389/fpsyg.2026.1872569

**Published:** 2026-08-31

**Authors:** Yan Zhang, Jinyan Huang, Jai Kumar

**Affiliations:** 1 Jiaxing Nanhu University, Jiaxing, China; 2 Jiangsu University, Zhenjiang, China; 3 Xiangnan University, Chenzhou, China

**Keywords:** EFL speaking, evaluative judgment, feedback literacy, feedback uptake, managing affect, mixed methods, PLS-SEM

## Abstract

Feedback literacy concerns learners' capacities and dispositions to recognize the value of feedback, make evaluative judgments, manage affective responses, and take action. This quantitatively dominant mixed-methods study examined theory-informed associations among these four features using questionnaire data from 2,116 first- and second-year Chinese English majors, two student open-ended items, and interviews with 18 instructors. Data analysis used reflective Mode A partial least squares path modeling, 5,000 bootstrap resamples, and 10-fold PLSpredict repeated 10 times. All four hypothesized paths were positive (beta = 0.457–0.830, *p* < 0.001), with *R*^2^ values of 0.532–0.730. However, HTMT values ranged from 0.921 to 1.022 and all exceeded 0.90, indicating inadequate discriminant validity. SRMR was 0.071 for the saturated model and 0.079 for the estimated model. Q2_predict values were positive (0.257–0.531), but the linear benchmark had lower RMSE for 13 of 14 indicators, indicating limited out-of-sample predictive advantage. Qualitative themes emphasized targeted practice, self-assessment, feedback seeking, actionable comments, affective support, and structured peer dialogue. The findings support an intertwined rather than fixed developmental view of speaking-feedback literacy and require cautious interpretation because of construct overlap and cross-sectional self-report data.

## Introduction

Speaking is a socially and cognitively demanding form of second-language performance. Learners must formulate ideas, retrieve linguistic resources, monitor accuracy, manage fluency, respond to interlocutors, and regulate anxiety in real time (Goh and Burns, 2012; Hughes, 2002; Luoma, 2004). For English-as-a-foreign-language (EFL) learners, limited opportunities for sustained interaction and concern about public error can make feedback particularly consequential. Comments on pronunciation, lexis, grammar, fluency, interaction, or organization may help learners notice gaps, but the presence of feedback alone does not ensure that learners understand, trust, or use it.

Feedback in speaking classrooms includes, but is not limited to, oral corrective feedback (OCF). OCF refers specifically to responses to learner error during or after oral production, whereas feedback literacy refers to learners' capacities to make sense of and use information from multiple sources, including instructors, peers, self-assessment, recordings, rubrics, automated tools, and task outcomes (Carless and Boud, 2018; Winstone et al., 2017). Keeping these concepts distinct is important: evidence that a particular corrective technique supports language development does not by itself show that learners possess the judgment, emotional regulation, and agency needed to convert that information into subsequent practice.

Carless and Boud (2018) conceptualized student feedback literacy through four interrelated features: appreciating feedback (AF), making judgments (MJ), managing affect (MA), and taking action (TA). The framework deliberately shifts attention from feedback delivery to learner uptake. Recent measurement studies have reinforced the importance of rigorous construct specification. Generic and behavior-oriented instruments have produced different dimensional structures, and recent EFL-specific validation work has emphasized disciplinary contextualization, expert review, independent psychometric testing, and sensitivity to change (Dawson et al., 2024; Teng and Ma, 2024; Woitt et al., 2025; Zhan, 2022; Zhu et al., 2025). These developments caution against treating any contextualized item set as a validated scale merely because its internal-consistency coefficients are acceptable.

Empirical work in EFL settings has concentrated mainly on writing, where feedback use can be traced through visible revisions. Longitudinal and intervention studies indicate that appreciation, judgment, affect, and action may develop unevenly and interactively (Weng et al., 2024; Zhang et al., 2023, 2025). Speaking presents a complementary context because uptake may involve repeated rehearsal, targeted pronunciation work, interactional practice, or changes in planning and monitoring rather than a single observable revision. Technology-supported peer feedback in EFL speaking also highlights the importance of motivation, participation, and opportunities to apply judgments in subsequent oral work (Ding and Zhu, 2025).

The present study revisits a large cross-sectional dataset of first- and second-year Chinese English majors and 18 instructors. It examines theory-informed associations among the four feedback-literacy features and treats them as interconnected rather than as an ordered pathway. The model arrows represent hypothesized contemporaneous associations within this sample; they do not establish temporal precedence or causality. The study also documents student-reported actions and instructor-reported supports and integrates these qualitative accounts with the quantitative pattern.

## A review of the literature

### Feedback, oral corrective feedback, and feedback literacy

Feedback has been described both as information about the gap between current and desired performance and as a process in which learners generate, interpret, discuss, and use information (Boud and Molloy, 2013; Hattie and Timperley, 2007; Yang and Carless, 2013). The process view is especially important in speaking because a comment such as “work on fluency” is too general to guide action unless the learner can diagnose what disrupted fluency, identify a feasible practice task, and evaluate whether later performance improved.

OCF research addresses the timing, explicitness, and interactional form of responses to language error (Ellis, 2009; Li, 2010; Lyster and Saito, 2010; Nassaji, 2016). Feedback-literacy research asks a different question: what enables learners to recognize useful information, judge standards and sources, regulate reactions, and act? The current questionnaire contains references to teacher, peer, self, and digital feedback; it therefore operationalizes a broader learner-side orientation rather than the effectiveness of a specific OCF technique. Accordingly, the study does not compare explicit with implicit feedback, immediate with delayed feedback, or teacher with peer feedback.

### The four interrelated features

#### Appreciating feedback (AF)

AF involves recognizing the potential value of feedback, understanding that information can come from multiple sources and forms, and seeing feedback as part of an ongoing learning process rather than as a terminal judgment (Carless and Boud, 2018). Appreciation may encourage engagement, but positive attitudes alone do not guarantee accurate judgment or action.

#### Making judgments (MJ)

MJ concerns evaluative judgment: comparing oral work with criteria, exemplars, peers, or prior performance and identifying the nature of a gap. In speaking, this can involve distinguishing a pronunciation problem from a fluency, lexical, grammatical, interactional, or organizational problem. Peer review and self-assessment can create opportunities to exercise this capacity, but learners need shared criteria and guided practice.

#### Managing affect (MA)

MA refers to responding productively to disappointment, criticism, embarrassment, or defensiveness. Speaking feedback can be identity-relevant because performance is public and time-pressured. Affect management does not mean suppressing emotion; it means maintaining sufficient psychological safety and self-regulation to interpret feedback and choose a response.

#### Taking action (TA)

TA is the behavioral enactment of feedback: seeking clarification, rehearsing, revising a plan, practicing a sound or discourse feature, recording and comparing performances, or applying feedback in a later task. Action is central to feedback value, but it also depends on opportunity, time, instructional design, and learner goals.

### Recent measurement and EFL research

Recent scale-development studies demonstrate that feedback literacy is not measured identically across frameworks. Zhan (2022) identified six dimensions in a generic student feedback literacy scale. Dawson et al. (2024) developed a behavior-focused measure organized around seeking, making sense of, using, providing, and managing affect. Woitt et al. (2025) reported two broad dimensions—feedback attitudes and feedback practices—after exploratory factor and Rasch analyses. In EFL-specific research, Teng and Ma (2024) validated a metacognition-based scale for academic writing, while Zhu et al. (2025) developed an EFL-context scale through a formal validation process. Together, these studies show that acceptable alpha, composite reliability, and average variance extracted values are necessary but not sufficient evidence of construct validity.

EFL feedback-literacy development also appears situated and non-linear. Zhang et al. (2023) documented cognitive, behavioral, and emotional development through longitudinal case analysis. Weng et al. (2024) found that a semester of peer feedback improved appreciation and judgment but not all other dimensions. Farangi et al. (2025) further showed that learner characteristics can shape preferences for written corrective feedback, reinforcing the need to interpret group patterns cautiously rather than essentialize demographic differences. Although much of this evidence comes from writing, it supports a broader conclusion relevant to speaking: feedback literacy is likely to be context-sensitive, developed through practice, and imperfectly captured by one-time self-report measures.

Teacher practices form part of the ecology in which student feedback literacy develops. Xie and Liu (2024) found that university EFL teachers' feedback-enabling practices included cognitive steering, behavioral regulation, and affective support, even when cultivation was not systematically planned. This provides a rationale for including instructor interviews in the present study: teachers' accounts do not validate the student path model, but they can contextualize the opportunities and supports through which students may interpret and act on speaking feedback.

### Theory-informed associations and alternative explanations

Carless and Boud (2018) describe AF, MJ, MA, and TA as interrelated features. The directional paths specified here are context-specific, theory-informed hypotheses about associations and are not intended to represent an ordered developmental process. AF may be positively associated with MJ because learners who value feedback may be more willing to compare performances and engage with criteria. AF may also be associated with MA because interpreting criticism as useful information may reduce threat. MJ may be associated with MA because a specific diagnosis can make negative feedback more manageable than a global judgment. MA may be associated with TA because discouragement and defensiveness can interrupt follow-up behavior.

These propositions are not exclusive. Action may increase appreciation after learners experience improvement; repeated practice may strengthen judgment; and clearer judgment may sometimes heighten anxiety by making weaknesses more salient. Strong positive associations may also reflect a general feedback orientation, self-efficacy, learner autonomy, social desirability, acquiescent responding, or common method variance. The cross-sectional design cannot adjudicate among these alternatives.

### Research questions and hypotheses

**Figure 1:**
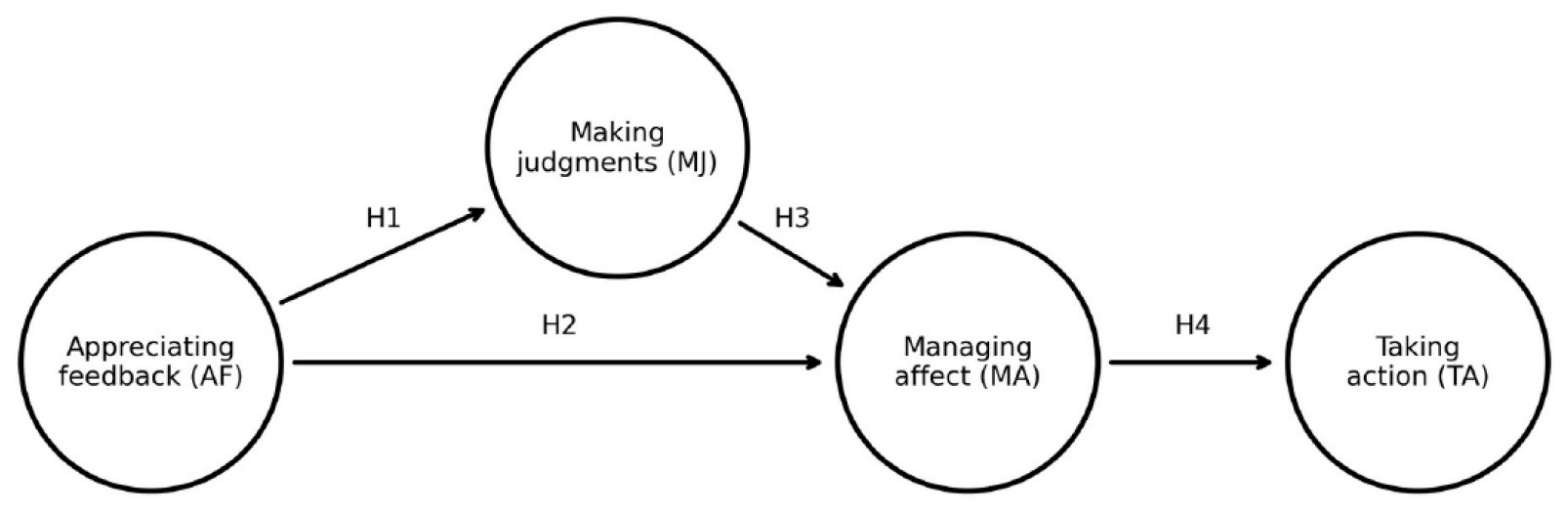
Theory-informed model of the four proposed feedback-literacy features. AF, appreciating feedback; MJ, making judgments; MA, managing affect; TA, taking action. The arrows represent theory-informed associations specified for statistical testing; no temporal or developmental ordering is implied.

RQ1: What theory-informed structural associations are observed among AF, MJ, MA, and TA in this cross-sectional sample?
RQ2: What actions do first- and second-year Chinese English majors report taking after speaking feedback, and what practices do college English instructors report using to support such action?
H1: AF is positively associated with MJ.
H2: AF is positively associated with MA.
H3: MJ is positively associated with MA.
H4: MA is positively associated with TA (see Figure 1).

## Method

### Research design

The study used a quantitatively dominant mixed-methods design. The quantitative strand consisted of a student questionnaire. The qualitative strand comprised two student open-ended items embedded in the survey and semi-structured interviews with 18 instructors. Integration occurred during interpretation through a joint display that compared the structural pattern with reported actions and instructional supports (Fetters et al., 2013). Because the instructor and student samples were not designed as matched dyads, instructor accounts are treated as contextual evidence rather than direct explanations of particular students' scores.

### Participants and sampling

A total of 2,262 first- and second-year English majors responded to the online survey. The analysis excluded 146 records solely because the same response option was selected for every questionnaire item, leaving the final dataset of 2,116 cases (see Table 1). Invariant responding was the only documented response-quality screening criterion; the retained records do not contain completion-time thresholds, attention checks, duplicate detection, or additional diagnostics for implausible response patterns.

**Table 1:** Sample characteristics.

Characteristic	Category	*n*	%
Gender	Men	315	14.9
Gender	Women	1,801	85.1
Year	First year	1,469	69.4
Year	Second year	647	30.6
TEM-4 status	Passed	36	1.7
TEM-4 status	Not passed	2,080	98.3
Total		2,116	100.0

The survey link was circulated through WeChat groups with assistance from professors and advisers. The sample is therefore a large convenience sample, not a nationally representative sample. The marked predominance of women and first-year students further limits subgroup and population-level inference.

A total of 104 public universities were involved, representing 21 provinces across China. Approximately 3,000 students were invited to participate, of whom 2,262 completed the survey, yielding a response rate of 75.4%. Data collection took place from October to December 2024.

Eighteen college English instructors were recruited through convenience sampling for online semi-structured interviews. Twelve were women and six were men; 13 held graduate degrees and five held undergraduate degrees. Twelve had more than 10 years of college-level EFL-speaking teaching experience and six had fewer than 10 years.

### Student questionnaire

The questionnaire was a researcher-developed contextual operationalization of the four features proposed by Carless and Boud (2018); it should not be described as a scale created or validated by those authors. The questionnaire contained 20 five-point Likert items (1 = strongly disagree to 5 = strongly agree): five AF items, four MJ items, six MA items, and five TA items. Generic feedback concepts were contextualized by referring to English speaking, speaking homework, feedback from teachers and peers, digital tools, judgments about oral work, reactions to negative feedback, and follow-up action (see Appendix A).

The questionnaire was initially developed in English and subsequently translated into Chinese for data collection. A forward- and back-translation procedure was used to ensure the accuracy and equivalence of the Chinese version (Sperber et al., 1994). Two professors specializing in English-Chinese translation independently translated the questionnaire into Chinese, and two additional translation professors translated the Chinese version back into English for comparison with the original version. The items were then reviewed by three experts. Before formal administration, the English version of the instrument was pilot tested with 100 Chinese English majors to assess the clarity, comprehensibility, wording, and linguistic appropriateness of the items. Minor wording adjustments were made based on the pilot-test feedback. The pilot testing was intended to improve the comprehensibility and linguistic appropriateness of the instrument and was not treated as formal validation of the questionnaire.

The instrument is treated as a preliminary contextual measure. This qualification is especially important because MA1–MA4 concern help seeking and continuous improvement more directly than emotion regulation, creating potential content overlap with AF and TA.

In a 20-item reanalysis, AF3 (digital access/storage) loaded 0.473 and TA4 (self-regulation) loaded 0.380. They were therefore excluded from the model. Removing AF3 narrows representation of digitally mediated feedback, whereas removing TA4 narrows self-regulatory action. Both items remain listed in Appendix A and are clearly identified as excluded; the statistical improvement does not eliminate the conceptual cost of deletion.

### Student open-ended items and instructor interview guide

Two open-ended items were included to elicit actions students reported taking after receiving feedback on speaking. Furthermore, the instructor on-line interviews addressed ways of making feedback specific and actionable, creating psychologically supportive speaking opportunities, encouraging self-assessment and reflection, and using peer feedback (see Appendix B).

### Data collection

The questionnaire was administered through the Survey Star platform and distributed via university-related WeChat groups. Participants received study information emphasizing the voluntary nature of participation and the confidentiality of their responses. Following convenience recruitment, the instructor interviews were conducted asynchronously via email: instructors received the interview questions by email and returned their written responses to the researchers by email.

### Quantitative analysis

The raw data were independently analyzed with a Mode A partial least squares path-modeling routine using the path-weighting scheme and standardized indicators. The analysis produced the item loadings to within 0.003, supporting computational comparability while allowing the diagnostics to be calculated. Reflective specification assumes that the indicators are manifestations of an underlying domain and should share variance; this assumption remains contestable for a heterogeneous item set that includes valuing feedback, help seeking, affect regulation, and action.

PLS-SEM was conducted for the exploratory and prediction-oriented analysis and its integration with NCA (Hair et al., 2022; Henseler et al., 2015, 2016). It does not convert cross-sectional associations into causal effects. Given the large sample and explicitly specified theory, covariance-based confirmatory factor analysis and comparison of alternative measurement structures would also be appropriate and are recommended for future validation.

The final workbook contained no missing values. Four MJ2 responses were coded 6 although the stated scale ranged from 1 to 5; these values were recoded to the valid maximum of 5. A sensitivity analysis retaining the four values of 6 changed no path by more than 0.001, no loading by more than 0.003, and no HTMT estimate by more than 0.001. The retained 18-item model was evaluated using loadings, Cronbach's alpha, composite reliability, AVE, the Fornell-Larcker criterion (Fornell and Larcker, 1981), HTMT with 5,000 percentile-bootstrap confidence intervals, saturated and estimated SRMR, inner and full-collinearity VIFs (Kock, 2015), and a one-factor exploratory check for common method concentration. No *post hoc* diagnostic was treated as capable of ruling out common method bias.

The structural model yielded four pre-specified direct associations. Standardized paths, *R*^2^, *f*^2^, and 5,000-resample bootstrap standard errors and confidence intervals were reported. Products of coefficients were calculated as statistical indirect associations, not temporal mediation mechanisms. Out-of-sample prediction was assessed with PLSpredict using 10 folds and 10 repetitions; item-level Q2_predict, RMSE, and MAE were compared with a direct-antecedent linear-model benchmark (Shmueli et al., 2019). The gender multigroup analysis remained excluded because the groups were markedly unbalanced and a MICOM assessment was not undertaken; no claim of gender effectiveness is warranted.

### Exploratory necessary condition analysis

Necessary condition analysis (NCA) was run from the 2,116-case raw dataset using the re-estimated construct scores from the retained 18-item reflective Mode A PLS model (Richter et al., 2020). The four MJ2 values recorded as 6 were recoded to the valid maximum of 5, consistent with the quantitative analysis described above. Because the PLS construct scores had many distinct observed values (101–273 across AF, MJ, MA, and TA) and were therefore treated as approximately continuous, Ceiling Regression-Free Disposal Hull (CR-FDH) was selected as the primary ceiling-line method. The analysis examined the upper-left necessity corner and used the empirical scope defined by the observed minima and maxima of each condition and outcome. Statistical significance was evaluated with 10,000 approximate permutation resamples for each condition-outcome pair (random seed = 20260813), in which outcome values were randomly permuted relative to the condition. Effect size *d* was calculated as the ceiling-zone area divided by the empirical scope. For descriptive interpretation, Dul (2020) general benchmarks were used: 0 < *d* < 0.10 = small, 0.10 < = *d* < 0.30 = medium, 0.30 < = *d* < 0.50 = large, and *d* > = 0.50 = very large; a 95% ceiling-accuracy value was used as a general descriptive reference. Ceiling accuracy, condition inefficiency, and outcome inefficiency were also examined. The permutation procedure follows Dul et al. (2020). NCA remains a supplementary exploratory analysis and is not interpreted as evidence of temporal precedence, developmental status, or causal necessity.

### Qualitative analysis

The open-ended responses and instructor interviews were analyzed through qualitative content analysis with inductive thematic categorization. The researchers initially color-coded, sorted, and organized responses and then met to consolidate recurring themes. Consensus review was used to reconcile coding decisions (see Appendix C). However, the intercoder reliability was not calculated.

Both the questionnaire for English majors and the interview questions for English instructors were administered in English. The open-ended responses and instructor interview responses were first entered into a spreadsheet and subsequently analyzed using a hybrid thematic approach at the level of each participant's response to each open-ended or interview question. One researcher conducted the initial coding by identifying meaning units and applying both deductive codes informed by the conceptual framework and inductive codes reflecting recurring ideas. A second researcher reviewed and refined the codes and candidate themes. Overlapping codes were permitted when a response contained more than one analytically relevant idea, and coding differences were resolved through discussion and refinement of the code definitions.

### Mixed-methods integration

Integration was conducted at the interpretation stage. A joint display aligns the quantitative domains with student actions and instructor supports, while also identifying non-convergence and methodological cautions. The qualitative strand does not validate path coefficients. Its role is to show how participants described processes that may correspond to the four conceptual features and where those descriptions cut across the proposed boundaries.

### Ethical considerations

The study was approved by the Research Ethical Review Board of the corresponding author's research institution. Participants received information about voluntariness and confidentiality and provided informed consent for participation and anonymized reporting.

## Results

### Measurement model

The item loadings ranged from 0.692 to 0.803. Cronbach's alpha ranged from 0.776 to 0.834, composite reliability from 0.856 to 0.879, and AVE from 0.548 to 0.625 (Table 2). These results indicate internal consistency and convergent validity for the indicators in this sample. They do not establish content validity or discriminant validity. In the 20-item model, AF3 loaded 0.473 and TA4 loaded 0.380; their exclusion should therefore be interpreted jointly with the resulting loss of digital-feedback and self-regulation content.

**Table 2:** Reliability and convergent-validity results for retained indicators.

Constructs	Items	Loading	alpha	CR	AVE
AF	AF1	0.761	0.776	0.856	0.598
	AF2	0.788			
	AF4	0.785			
	AF5	0.759			
MJ	MJ1	0.800	0.798	0.869	0.625
	MJ2	0.803			
	MJ3	0.761			
	MJ4	0.798			
MA	MA1	0.696	0.834	0.879	0.548
	MA2	0.782			
	MA3	0.749			
	MA4	0.764			
	MA5	0.754			
	MA6	0.692			
TA	TA1	0.798	0.784	0.860	0.607
	TA2	0.745			
	TA3	0.794			
	TA5	0.778			

AF, appreciating feedback; MJ, making judgments; MA, managing affect; TA, taking action; CR, composite reliability; AVE, average variance extracted. AF3 and TA4 were excluded after loading 0.473 and 0.380, respectively, in the full 20-item reanalysis.

### Discriminant validity and construct overlap

The Fornell-Larcker criterion was not satisfied (see Table 3). The square root of AVE for AF (0.773) was lower than its correlations with MA (0.794) and TA (0.796); the MJ diagonal (0.791) was slightly lower than the MJ-MA correlation (0.795); the MA diagonal (0.740) was lower than its correlations with AF (0.794), MJ (0.795), and TA (0.830); and the TA diagonal (0.779) was lower than its correlations with AF (0.796), MJ (0.782), and MA (0.830).

**Table 3:** Fornell-Larcker matrix.

Construct	AF	MJ	MA	TA
AF	0.773			
MJ	0.730	0.791		
MA	0.794	0.795	0.740	
TA	0.796	0.782	0.830	0.779

Diagonal entries are square roots of AVE; off-diagonal entries are construct-score correlations. Boldface is not used because the criterion fails for multiple pairs.

HTMT provided a stricter confirmation of the overlap (see Table 4). Estimates ranged from 0.921 to 1.022; every pair exceeded the 0.90 guideline. Bootstrap intervals approached or included 1.00 for several pairs, and the MA-TA interval was entirely above 1.00. The four domains therefore cannot be treated as clearly discriminant on the basis of this instrument, even though each indicator loaded most strongly on its intended composite. Accordingly, subsequent structural coefficients are interpreted as associations among overlapping composites rather than as relationships among clearly separable latent dimensions.

**Table 4:** HTMT ratios with 5,000-resample percentile-bootstrap confidence intervals.

Construct pair	HTMT	95% CI	Assessment
AF-MJ	0.921	[0.893, 0.950]	Exceeds 0.90
AF-MA	0.981	[0.958, 1.003]	Exceeds 0.90
AF-TA	1.018	[0.997, 1.041]	Exceeds 0.90
MJ-MA	0.968	[0.947, 0.988]	Exceeds 0.90
MJ-TA	0.985	[0.963, 1.009]	Exceeds 0.90
MA-TA	1.022	[1.007, 1.037]	Exceeds 0.90

HTMT, heterotrait-monotrait ratio. All estimates exceed 0.90; bootstrap intervals are percentile intervals.

### Model fit, collinearity, and common method diagnostics

The saturated SRMR was 0.071 and the estimated-model SRMR was 0.079, both at or just below the commonly used 0.08 reference value. Inner VIFs ranged from 1.000 to 2.138, whereas full-collinearity VIFs ranged from 3.189 to 4.332 (see Table 5). The first unrotated factor from all 20 questionnaire items accounted for 45.89% of variance. These diagnostics do not indicate extreme structural collinearity under a VIF < 5 rule, but the full VIFs, high HTMT values, and common administration format leave substantial concern about construct redundancy and common method variance.

**Table 5:** Model fit, collinearity, and common method diagnostics.

Diagnostic	Result	Interpretation
SRMR, saturated	0.071	Below 0.08 reference
SRMR, estimated	0.079	Near 0.08 reference
Inner VIF range	1.000–2.138	No severe predictor collinearity
Full-collinearity VIF range	3.189–4.332	Some values exceed 3.3 heuristic
First unrotated factor	45.89%	Below 50%, but not a definitive bias test

SRMR, standardized root mean square residual; VIF, variance inflation factor. No single *post hoc* statistic can exclude common method bias.

### Theory-informed structural associations

**Figure 2:**
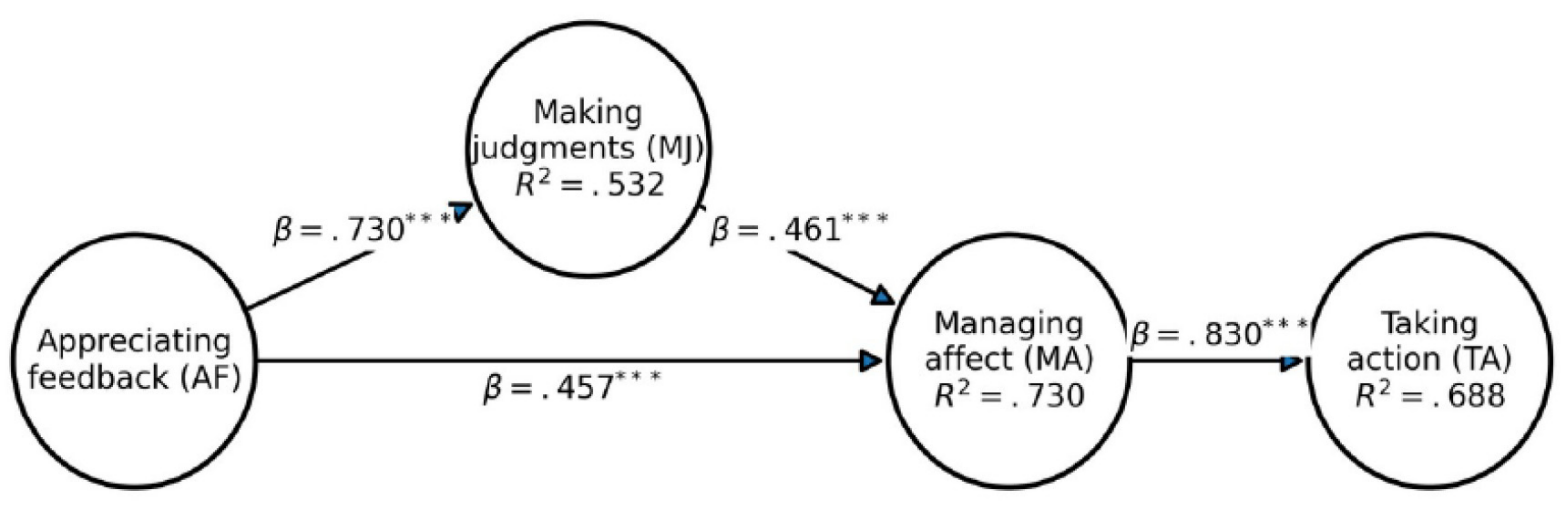
Estimated structural associations. ***p < 0.001. Arrows represent model-implied contemporaneous associations only.

All four standardized paths were positive and statistically different from zero, with beta values from 0.457 to 0.830 (see Table 6 and Figure 2). The estimated MA-to-TA coefficient was 0.830 (SE = 0.008, *t* = 108.279, 95% CI [0.815, 0.844]). The model accounted for 53.2% of the variance in MJ, 73.0% in MA, and 68.8% in TA. Given the failed discriminant-validity tests, these coefficients describe associations within the specified composite model and should not be interpreted as evidence of distinct mechanisms.

**Table 6:** Direct structural associations.

H	Path	beta	SE	*t*	95% CI	*f* ^2^	Outcome *R*^2^
H1	AF->MJ	0.730	0.013	57.214	[0.704, 0.754]	1.138	0.532
H2	AF->MA	0.457	0.019	23.994	[0.420, 0.495]	0.362	0.730
H3	MJ->MA	0.461	0.020	23.365	[0.423, 0.500]	0.368	0.730
H4	MA->TA	0.830	0.008	108.279	[0.815, 0.844]	2.210	0.688

Bootstrap resamples = 5,000; all *p* < 0.001. beta, standardized path coefficient; SE, bootstrap standard error; *f*^2^, local effect size; *R*^2^ is repeated for paths sharing the MA outcome. Associations are not causal or temporal effects.

### Bootstrapped indirect associations

The coefficient products were also positive, and their bootstrap intervals excluded zero (see Table 7). Because all measures were collected at one time and the constructs were insufficiently discriminant, these products are reported only as indirect statistical associations. They do not establish temporal mediation or a mechanism through which one feature changes another.

**Table 7:** Bootstrapped indirect statistical associations.

Path	Estimate	SE	*t*	95% CI
AF -> MA -> TA	0.379	0.017	22.820	[0.347, 0.412]
MJ -> MA -> TA	0.383	0.017	22.393	[0.350, 0.416]
AF -> MJ -> MA	0.337	0.015	22.118	[0.307, 0.367]
AF -> MJ -> MA -> TA	0.279	0.014	20.683	[0.253, 0.306]

All *p* < 0.001 based on 5,000 bootstrap resamples. These are products of contemporaneous path coefficients, not evidence of temporal mediation.

### Out-of-sample predictive assessment

All item-level Q2_predict values were positive, ranging from 0.257 to 0.531, indicating that the PLS predictions improved on a training-mean benchmark (see Table 8). However, the direct-antecedent linear model produced lower RMSE for 13 of 14 indicators; the only PLS advantage was a negligible RMSE difference for MJ2. Under PLSpredict guidance, this pattern indicates low predictive power relative to the linear benchmark despite moderate in-sample *R*^2^ values.

**Table 8:** PLSpredict results: 10-fold cross-validation repeated 10 times.

Item	Q2_predict	PLS RMSE	LM RMSE	Difference	PLS MAE	LM MAE
MJ1	0.293	0.771	0.769	0.003	0.622	0.624
MJ2	0.359	0.667	0.667	−0.000	0.514	0.514
MJ3	0.257	0.808	0.807	0.002	0.645	0.647
MJ4	0.409	0.636	0.632	0.004	0.492	0.489
MA1	0.330	0.767	0.758	0.009	0.605	0.589
MA2	0.531	0.588	0.581	0.007	0.418	0.401
MA3	0.382	0.704	0.696	0.008	0.516	0.506
MA4	0.409	0.725	0.700	0.024	0.533	0.505
MA5	0.405	0.688	0.683	0.005	0.496	0.493
MA6	0.329	0.770	0.754	0.016	0.568	0.561
TA1	0.431	0.659	0.643	0.015	0.532	0.518
TA2	0.356	0.712	0.699	0.013	0.554	0.547
TA3	0.432	0.655	0.653	0.002	0.474	0.467
TA5	0.446	0.624	0.608	0.016	0.451	0.439

LM, direct-antecedent linear-model benchmark. Difference, PLS RMSE—LM RMSE; negative values favor PLS. Q2_predict was positive for every indicator, but PLS had lower RMSE only for MJ2, by less than 0.001.

### Exploratory NCA results

The CR-FDH run yielded *d* = 0.2464 for AF-MJ, *d* = 0.3178 for AF-MA, *d* = 0.2813 for MJ-MA, and *d* = 0.2687 for MA-TA (see Table 9). None of the 10,000 permuted effect sizes equaled or exceeded the corresponding observed effect, producing *p* = 0.0001 for each condition-outcome pair under the permutation-test convention. Using the unrounded estimates and Dul (2020) general benchmarks, AF-MJ, MJ-MA, and MA-TA were medium effects, whereas AF-MA was a large effect. Ceiling accuracy ranged from 97.50% to 99.20%, exceeding the 95% descriptive reference for all four pairs. Condition inefficiency ranged from 14.26% to 47.65%, and outcome inefficiency ranged from 0.00% to 37.45%. These quantities describe ceiling constraints within the observed score ranges and should not be interpreted as temporal stages or universal pedagogical thresholds.

**Table 9:** CR-FDH NCA results and diagnostic statistics.

Condition-outcome	*d*	Permutation *p*	Accuracy (%)	Cond. ineff. (%)	Outcome ineff. (%)
AF-MJ	0.2464	0.0001	99.20	21.21	37.45
AF-MA	0.3178	0.0001	97.50	14.26	25.87
MJ-MA	0.2813	0.0001	98.96	47.65	0.00
MA-TA	0.2687	0.0001	98.96	25.49	27.87

NCA, necessary condition analysis; CR-FDH, Ceiling Regression-Free Disposal Hull. Permutation test = 10,000 resamples per condition-outcome pair (random seed = 20260813). Effect-size categories are based on unrounded *d* values. Condition inefficiency is the percentage of the observed condition range in which the condition does not constrain the outcome; outcome inefficiency is the percentage of the observed outcome range in which the outcome is not constrained by the condition.

### Student-reported actions after speaking feedback

The following four recurring themes were identified from the open-ended data analysis:

#### Further speaking practice

Students described seeking opportunities to use feedback in class discussions, presentations, role-plays, conversation clubs, or online speaking settings. A second-year student stated, “*I often choose to join conversation clubs or online speaking forums where I can interact with native English speakers to practice speaking in a supportive environment*.”

#### Targeted diagnosis and practice

Students described using feedback to identify pronunciation, intonation, fluency, vocabulary, grammar, or organization as a specific focus and then selecting a related exercise. A first-year student reported, “*I often practice pronunciation drills, listen to and repeat recordings of native speakers, or engage in language-learning apps that provide speaking exercises and feedback*.”

#### Self-assessment and reflection

Participants described reviewing a completed speaking task, comparing it with feedback, and setting a narrower goal. A first-year student explained, “*If I receive feedback that my pronunciation is unclear, I then focus on practicing specific sounds or words until I achieve greater clarity in my speech*.”

#### Feedback seeking

Students also described asking instructors or peers for clarification, arranging additional consultation, or recording speech for review. A second-year student stated, “*By actively seeking out feedback and incorporating it into my practice, I can take proactive steps toward improving my English speaking ability*.”

### Instructor-reported practices intended to support action

The following four recurring themes were identified from the interview data analysis.

#### Specific and actionable feedback

All 18 instructors were reported as emphasizing timely, constructive comments connected to a concrete follow-up task. One experienced instructor stated, “*If my students struggle with pronunciation, I often provide targeted feedback on specific sounds or word-stress patterns, along with practice exercises to help improve their pronunciation*.”

#### Psychologically supportive practice opportunities

Instructors described role-plays, debates, presentations, and other speaking opportunities in a non-judgmental environment. These practices align most directly with MA and TA by reducing threat and providing an immediate opportunity to try again.

#### Self-assessment and reflection

Half of the instructors emphasized checklists, rubrics, recordings, or reflective prompts. These practices connect MJ with TA by helping learners compare performance, identify a target, and plan follow-up.

#### Peer feedback and collaborative learning

Eight instructors explicitly recommended structured peer feedback. One early-career instructor stated, “*By engaging in peer feedback exchanges, my students can gain valuable insights into their own speaking abilities and learn from their classmates' strengths and weaknesses*.”

### Mixed-methods joint display

Table 10 is a mixed-methods joint display. Integration of quantitative patterns with qualitative themes shows convergence around interpretation, affective support, and action opportunities; whereas identifying where the qualitative evidence does not resolve measurement or predictive limitations.

**Table 10:** Integration of quantitative patterns with qualitative themes.

Quantitative pattern	Student-reported evidence	Instructor-reported evidence	Integrated interpretation
AF-MJ association (beta = 0.730)	Students sought and revisited feedback to identify targets	Teachers used criteria, examples, and specific diagnosis	Valuing feedback may co-occur with judgment, but HTMT = 0.921 indicates overlap
AF/MJ-MA associations (beta = 0.457/0.461)	Students reframed weaknesses as practice goals	Teachers emphasized supportive, non-judgmental environments	Accounts show affect and judgment as reciprocally related
MA-TA association (beta = 0.830)	Students described persistence, rehearsal, and targeted practice	Teachers linked comments to immediate follow-up opportunities	Context supports an affect-action link, but HTMT = 1.022 limits distinct-construct interpretation
Positive Q2_predict; weak LM comparison	No account established out-of-sample predictive accuracy	No instructor account established prediction benchmarks	Qualitative evidence contextualizes practices but cannot validate predictive performance
NCA effects	No student account established universal thresholds	No instructor account established universal prerequisites	NCA remains exploratory; qualitative data do not validate bottleneck thresholds

## Discussion

### Principal findings and theoretical interpretation

The study produced four main findings. First, the four questionnaire scores were strongly positively associated, but both Fornell-Larcker and HTMT analyses showed that their empirical distinctness was inadequate. Second, the PLS analysis yielded positive standardized paths from 0.457 to 0.830, with the MA-to-TA association estimated at beta = 0.830. Third, all Q2_predict values were positive, but the PLS model rarely outperformed a direct-antecedent linear benchmark, indicating limited out-of-sample predictive advantage. Fourth, students and instructors described feedback use as intertwined practices involving diagnosis, emotion, opportunity, reflection, seeking, and repeated speaking activity.

The AF-MJ path (beta = 0.730) is consistent with the proposition that learners who recognize feedback's value may be more willing to examine criteria and compare performance. Yet HTMT was 0.921, and the AF score correlated 0.794 with MA and 0.796 with TA. These results suggest that the scores partly capture a broad positive feedback orientation rather than cleanly separable capacities. Recent measurement research likewise shows that feedback-literacy dimensions depend on item framing and validation method (Dawson et al., 2024; Woitt et al., 2025; Zhu et al., 2025). The present findings support the conceptual relevance of appreciation, judgment, affect, and action, but not a definitive four-factor measurement claim.

The qualitative findings further support a reciprocal and iterative interpretation of the four features. Students often described action and judgment recursively: practicing generated new self-assessment information, and seeking feedback created additional opportunities to appreciate or question feedback. Teachers likewise described affective support, criteria, practice, and reflection as mutually reinforcing. This is more consistent with an ecological, iterative view of feedback literacy than with a fixed developmental ladder (Chong, 2021; Molloy et al., 2020).

### Managing affect and taking action

The MA-TA association is conceptually plausible because embarrassment, defensiveness, and discouragement can interrupt feedback uptake, especially in public speaking contexts. The estimated standardized path was 0.830, with *R*^2^ = 0.688 for TA. Student accounts of targeted practice and instructor accounts of supportive rehearsal provide contextual evidence that emotional safety and opportunity may work together. Xie and Liu (2024) study of Chinese university EFL teachers similarly identified affective support alongside cognitive and behavioral practices.

Nevertheless, the 0.830 coefficient cannot be treated as evidence that affect is a dominant or decisive mechanism. HTMT for MA and TA was 1.022, indicating that the two composites were not empirically distinct. The current study also did not measure momentary emotional states, observed uptake, or subsequent speaking performance. The defensible conclusion is narrower: self-reported affect-related and action-related tendencies were closely associated, and participants described practices that linked emotional support to follow-up behavior.

### Relationship to recent EFL feedback-literacy research

Recent longitudinal and intervention research has found uneven change across feedback-literacy dimensions. Weng et al. (2024) observed gains in appreciation and judgment after peer-feedback activities but not equivalent changes in all dimensions. Zhang et al. (2023) documented development through cognitive, emotional, and behavioral processes over time, while Zhang et al. (2025) emphasized iterative negotiation and revision in peer feedback. These studies caution against inferring development from cross-sectional path estimates and support the present revision's emphasis on reciprocal processes.

Farangi et al. (2025) showed that learner characteristics can shape feedback preferences, but preference differences do not establish superior feedback use. The gender comparison lacked MICOM invariance testing, used highly unequal groups, and offered no objective criterion of effectiveness. Future subgroup research should pre-specify hypotheses, provide inclusive category options, establish measurement invariance, report within-group estimates and confidence intervals, and adjust for multiple comparisons.

### Interpretation of the NCA

The fully specified CR-FDH rerun makes the NCA results reproducible but does not change their supplementary status. The four effect sizes ranged from 0.2464 to 0.3178 and were statistically unusual under 10,000 permutations, while ceiling accuracy ranged from 97.50% to 99.20%. The condition- and outcome-inefficiency diagnostics indicate portions of the observed score ranges where the fitted ceiling did not constrain the outcome or condition, respectively; they are sample-dependent boundary descriptors rather than instructional cutoffs. More importantly, the high HTMT values indicate substantial overlap among the four composites, so the NCA patterns cannot establish that one distinct feedback-literacy capacity is temporally or causally necessary for another. We therefore interpret NCA as a complementary description of the observed score-space constraints, not as evidence of developmental stages or universal prerequisites.

### Pedagogical implications

The implications should be read at three levels. Directly supported by the qualitative accounts, instructors can make speaking feedback more actionable by identifying a specific performance feature and linking it to a feasible next task; they can provide opportunities to rehearse, re-record, or repeat a task; and they can use reflection, self-assessment, and structured peer dialogue to help learners translate comments into goals.

Literature-informed but not directly tested in this study, instructors may normalize error, balance challenge with psychological safety, use exemplars and criteria to develop evaluative judgment, and prompt students to document what feedback they accepted, questioned, and acted on. Technology can support recording, replay, annotation, and distributed peer dialogue, but its contribution should be evaluated rather than assumed (Ding and Zhu, 2025).

Proposals such as emotional-intelligence workshops, personalized feedback plans, or mandated peer-review programs require experimental or design-based evaluation. The current study reports perceived practices; it does not show that any intervention improved feedback literacy or speaking proficiency. Future interventions should measure actual uptake, quality of subsequent speaking, retention over time, and possible differential effects across learners.

### Limitations and directions for future research

Several limitations must be acknowledged. First, the design was cross-sectional and relied primarily on one-time self-report data. Temporal ordering, reciprocal effects, mediation mechanisms, and causality cannot be established. Further, the study did not include objective ratings of oral performance, observed feedback uptake, feedback-source comparisons, or modality comparisons. Self-reported behavior may differ from actual practice.

Second, the questionnaire was a researcher-developed contextualization rather than a validated Carless and Boud scale. AF3 (0.473) and TA4 (0.380) were removed because of low factor loadings, and several MA items overlap conceptually with help seeking and action. These issues limit the evaluation of the instrument-development and cultural-adaptation process.

Third, discriminant validity was not established. HTMT estimates ranged from 0.921 to 1.022, and all exceeded 0.90. This substantial overlap limits interpretation of AF, MJ, MA, and TA as empirically distinct dimensions and requires the structural coefficients to be read as associations among overlapping composites. Alternative factor/composite models and independent-sample validation are needed before the four scores can be treated as distinct.

Fourth, all focal variables used the same response format at the same time. Full-collinearity VIFs ranged from 3.189 to 4.332, and the first unrotated factor accounted for 45.89% of item variance. These diagnostics do not rule out common method variance, acquiescence, social desirability, or a general positive response orientation.

Fifth, the sample was recruited through convenience sampling using university-related WeChat networks. Women and first-year students were overrepresented, and students may have been clustered within universities; however, no multilevel or design-effect assessment was conducted. Moreover, invariant responding was the only documented criterion used to exclude responses based on data quality.

Finally, although the NCA was conducted using a fully specified CR-FDH procedure, empirical scope, and 10,000 permutations, its effect sizes, accuracy, and inefficiency diagnostics remain sample-dependent and sensitive to measurement quality. In particular, the substantial HTMT overlap among AF, MJ, MA, and TA limits the strength of substantive interpretations of the observed necessity patterns.

Future research should develop or adopt a speaking-specific feedback-literacy instrument through explicit content mapping, expert review, cognitive interviews, pilot testing, confirmatory comparison, HTMT and measurement-invariance assessment, and replication in an independent sample. Longitudinal or cross-lagged designs can examine reciprocal development, while experimental studies can test whether structured opportunities for judgment, affective support, and follow-up action improve observed speaking performance. Predictive studies should preregister benchmarks and evaluate whether PLS, regularized, or other models generalize to new learners and institutions.

## Conclusion

This study offers a large-sample but methodologically bounded account of feedback literacy for EFL speaking among first- and second-year Chinese English majors. The four proposed features were strongly associated, yet HTMT and Fornell-Larcker results showed that they were not sufficiently distinct. Although all Q2_predict values were positive, the model showed little predictive advantage over linear benchmarks. Student and instructor accounts nevertheless illuminate how feedback use may be enacted through targeted practice, self-assessment, feedback seeking, supportive interaction, and concrete follow-up opportunities. The most defensible contribution is evidence that cognitive, affective, and behavioral aspects of speaking-feedback use are closely intertwined, rather than evidence of an ordered developmental process. Stronger measurement, longitudinal evidence, direct observation, and independent predictive validation are needed before claims about mechanisms, necessity, or effective interventions can be sustained.

## Data Availability

The raw data supporting the conclusions of this article will be made available by the authors, without undue reservation.

## References

[B1] Boud D. Molloy E. (2013). Rethinking models of feedback for learning: the challenge of design. Assess. Eval. High. Educ. 38, 698–712. doi: 10.1080/02602938.2012.691462

[B2] Carless D. Boud D. (2018). The development of student feedback literacy: enabling uptake of feedback. Assess. Eval. High. Educ. 43, 1315–1325. doi: 10.1080/02602938.2018.1463354

[B3] Chong S. W. (2021). Reconsidering student feedback literacy from an ecological perspective. Assess. Eval. High. Educ. 46, 92–104. doi: 10.1080/02602938.2020.1730765

[B4] Dawson P. Yan Z. Lipnevich A. Tai J. Boud D. Mahoney P. (2024). Measuring what learners do in feedback: the feedback literacy behaviour scale. Assess. Eval. High. Educ. 49, 348–362. doi: 10.1080/02602938.2023.2240983

[B5] Ding Y. Zhu J. (2025). Mobile based peer feedback in EFL speaking: learners' motivation, behavioral engagement in feedback provision, and achievement. Asian-Pac. J. Second Foreign Lang. Educ. 10:2. doi: 10.1186/s40862-024-00314-9

[B6] Dul J. (2020). Conducting Necessary Condition Analysis. London: Sage. doi: 10.4337/9781788976954.00008

[B7] Dul J. van der Laan E. Kuik R. (2020). A statistical significance test for necessary condition analysis. Organ. Res. Methods 23, 385–395. doi: 10.1177/1094428118795272

[B8] Ellis R. (2009). Corrective feedback and teacher development. L2 J. 1, 3–18. doi: 10.5070/L2.V1I1.9054

[B9] Farangi M. R. Rashidi N. Jokar M. (2025). Preferences for written corrective feedback among field-dependent and field-independent EFL learners: a mixed-methods investigation. Soc. Sci. Humanit. Open 12:101700. doi: 10.1016/j.ssaho.2025.101700

[B10] Fetters M. D. Curry L. A. Creswell J. W. (2013). Achieving integration in mixed methods designs: principles and practices. Health Serv. Res. 48, 2134–2156. doi: 10.1111/1475-6773.12117 24279835 PMC4097839

[B11] Fornell C. Larcker D. F. (1981). Evaluating structural equation models with unobservable variables and measurement error. J. Market. Res. 18, 39–50. doi: 10.1177/002224378101800104

[B12] Goh C. C. M. Burns A. (2012). Teaching Speaking: A Holistic Approach. Cambridge: Cambridge University Press.

[B13] Hair J. F. Hult G. T. M. Ringle C. M. Sarstedt M. (2022). A Primer on Partial Least Squares Structural Equation Modeling (PLS-SEM), 3rd Edn. Thousand Oaks: Sage. doi: 10.1007/978-3-030-80519-7

[B14] Hattie J. Timperley H. (2007). The power of feedback. Rev. Educ. Res. 77, 81–112. doi: 10.3102/003465430298487

[B15] Henseler J. Ringle C. M. Sarstedt M. (2015). A new criterion for assessing discriminant validity in variance-based structural equation modeling. J. Acad. Market. Sci. 43, 115–135. doi: 10.1007/s11747-014-0403-8

[B16] Henseler J. Ringle C. M. Sarstedt M. (2016). Testing measurement invariance of composites using partial least squares. Int. Market. Rev. 33, 405–431. doi: 10.1108/IMR-09-2014-0304

[B17] Hughes R. (2002). Teaching and Researching Speaking. Harlow: Pearson Education.

[B18] Kock N. (2015). Common method bias in PLS-SEM: a full collinearity assessment approach. Int. J. e-Collab. 11, 1–10. doi: 10.4018/ijec.2015100101

[B19] Li S. (2010). The effectiveness of corrective feedback in SLA: a meta-analysis. Lang. Learn. 60, 309–365. doi: 10.1111/j.1467-9922.2010.00561.x

[B20] Luoma S. (2004). Assessing Speaking. Cambridge: Cambridge University Press. doi: 10.1017/CBO9780511733017

[B21] Lyster R. Saito K. (2010). Oral feedback in classroom SLA: a meta-analysis. Stud. Second Lang. Acquis. 32, 265–302. doi: 10.1017/S0272263109990520

[B22] Molloy E. Boud D. Henderson M. (2020). Developing a learning-centred framework for feedback literacy. Assess. Eval. High. Educ. 45, 527–540. doi: 10.1080/02602938.2019.1667955

[B23] Nassaji H. (2016). Interactional feedback in second language teaching and learning: a synthesis and analysis of current research. Lang. Teach. Res. 20, 535–562. doi: 10.1177/1362168816644940

[B24] Richter N. F. Schubring S. Hauff S. Ringle C. M. Sarstedt M. (2020). When predictors of outcomes are necessary: guidelines for the combined use of PLS-SEM and NCA. Ind. Manag. Data Syst. 120, 2243–2267. doi: 10.1108/IMDS-11-2019-0638

[B25] Shmueli G. Sarstedt M. Hair J. F. Cheah J.-H. Ting H. Vaithilingam S. . (2019). Predictive model assessment in PLS-SEM: guidelines for using PLSpredict. *Eur. J*. Market. 53, 2322–2347. doi: 10.1108/EJM-02-2019-0189

[B26] Sperber A. Devellis R. Boehlecke B. (1994). Cross-cultural translation: methodology and validation. J. Cross Cult. Psychol. 25, 501–524. doi: 10.1177/0022022194254006

[B27] Teng M. F. Ma M. (2024). Assessing metacognition-based student feedback literacy for academic writing. Assess. Writ. 59:100811. doi: 10.1016/j.asw.2024.100811

[B28] Weng F. Chen S. Zhao C. G. (2024). Effects of peer feedback in English writing classes on EFL students' writing feedback literacy. Assess. Writ. 61:100874. doi: 10.1016/j.asw.2024.100874

[B29] Winstone N. E. Nash R. A. Parker M. Rowntree J. (2017). Supporting learners' agentic engagement with feedback: a systematic review and a taxonomy of recipience processes. Educ. Psychol. 52, 17–37. doi: 10.1080/00461520.2016.1207538

[B30] Woitt S. Weidlich J. Jivet I. Orhan Goksun D. Drachsler H. Kalz M. (2025). Students' feedback literacy in higher education: an initial scale validation study. Teach. High. Educ. 30, 257–276. doi: 10.1080/13562517.2023.2263838

[B31] Xie Z. Liu W. (2024). What matters in the cultivation of student feedback literacy: exploring university EFL teachers' perceptions and practices. Hum. Soc. Sci. Commun. 11:162. doi: 10.1057/s41599-024-02648-8

[B32] Yang M. Carless D. (2013). The feedback triangle and the enhancement of dialogic feedback processes. Teach. High. Educ. 18, 285–297. doi: 10.1080/13562517.2012.719154

[B33] Zhan Y. (2022). Developing and validating a student feedback literacy scale. Assess. Eval. High. Educ. 47, 1087–1100. doi: 10.1080/02602938.2021.2001430

[B34] Zhang F. Li D. Zhao Y. E. Zhao Y. (2025). A longitudinal exploration of EFL learners' peer feedback literacy development. Stud. Educ. Eval. 87:101522. doi: 10.1016/j.stueduc.2025.101522

[B35] Zhang F. Min H. T. He P. Chen S. Ren S. (2023). Understanding EFL students' feedback literacy development in academic writing: a longitudinal case study. Assess. Writ. 58:100770. doi: 10.1016/j.asw.2023.100770

[B36] Zhu F. Balloo K. Medland E. Hosein A. (2025). Developing and validating a scale for student feedback literacy in an EFL context. Cogent Educ. 12:2528431. doi: 10.1080/2331186X.2025.2528431

