# Supplementary material for: Feedback literacy for EFL speaking among first- and second-year Chinese English majors: structural associations and reported action strategies
Source: Front Psychol. 2026 Aug 31;17:1872569. doi: 10.3389/fpsyg.2026.1872569 (PMC13572703; doi:10.3389/fpsyg.2026.1872569)
Supplement: Supplementary file 1 [file Supplementary_file_1.docx]

**Appendix A**

**Researcher-developed questionnaire contextualized to EFL speaking**

| Item | Wording | Status/measurement note |
| --- | --- | --- |
| AF1 | I understand and value the role of feedback in improving my English speaking. | Retained |
| AF2 | I know that feedback about my English speaking can come from different sources. | Retained |
| AF3 | I use digital tools to access, store, and revisit feedback on my English speaking. | Excluded: loading < .50 |
| AF4 | I know that feedback about my English speaking can come in different forms. | Retained |
| AF5 | I understand and value the role of feedback in becoming an active English speaker. | Retained |
| MJ1 | I develop the ability to make sound judgments about my peers’ English speaking homework. | Retained |
| MJ2 | I develop the ability to make sound judgments about my own English speaking homework. | Retained |
| MJ3 | I participate actively in peer-feedback activities in English speaking. | Retained |
| MJ4 | I develop evaluative capacity in English speaking so that I can make more robust judgments. | Retained |
| MA1 | I proactively ask teachers for suggestions about my English speaking when needed. | Retained; help-seeking content |
| MA2 | I develop habits of continuous improvement based on self-feedback in English speaking. | Retained; overlaps with action |
| MA3 | I develop habits of continuous improvement based on teacher and peer feedback in English speaking. | Retained; overlaps with action |
| MA4 | I proactively ask peers for suggestions about my English speaking when needed. | Retained; help-seeking content |
| MA5 | I remain emotionally stable when receiving negative feedback on my English speaking. | Retained |
| MA6 | I avoid becoming defensive when receiving negative feedback on my English speaking. | Retained |
| TA1 | I develop strategies for acting on feedback in my English speaking. | Retained |
| TA2 | I make full use of feedback about my English speaking to become a more active speaker. | Retained |
| TA3 | I draw inferences from feedback about my English speaking to improve continuously. | Retained |
| TA4 | I use feedback to self-regulate my English speaking. | Excluded: loading < .50 |
| TA5 | I recognize the importance of taking action in response to feedback about my English speaking. | Retained |

*Note: AF = appreciating feedback; MJ = making judgments; MA = managing affect; TA = taking action.*
